# *rpoB* mutations conferring rifampicin-resistance affect growth, stress response and motility in *Vibrio vulnificus*

**DOI:** 10.1099/mic.0.000991

**Published:** 2020-11-13

**Authors:** Laura Cutugno, Jennifer Mc Cafferty, Jan Pané-Farré, Conor O’Byrne, Aoife Boyd

**Affiliations:** ^1^​ Discipline of Microbiology, School of Natural Sciences, National University of Ireland Galway, Galway, Ireland; ^2^​ Institute of Microbiology, University of Greifswald, D-17489 Greifswald, Germany; ^3^​ Center for Synthetic Microbiology (SYNMIKRO) & Department of Chemistry, Philipps-University Marburg, Hans-Meerwein-Strasse, C07, 35043 Marburg, Germany

**Keywords:** motility, osmotolerance, rifampicin resistance, rpoB, stress response, *Vibrio vulnificus*

## Abstract

Rifampicin is a broad-spectrum antibiotic that binds to the bacterial RNA polymerase (RNAP), compromising DNA transcription. Rifampicin resistance is common in several microorganisms and it is typically caused by point mutations in the gene encoding the β subunit of RNA polymerase, *rpoB*. Different *rpoB* mutations are responsible for various levels of rifampicin resistance and for a range of secondary effects. *rpoB* mutations conferring rifampicin resistance have been shown to be responsible for severe effects on transcription, cell fitness, bacterial stress response and virulence. Such effects have never been investigated in the marine pathogen *Vibrio vulnificus *, even though rifampicin-resistant strains of *V. vulnificus* have been isolated previously. Moreover, spontaneous rifampicin-resistant strains of *V. vulnificus* have an important role in conjugation and mutagenesis protocols, with poor consideration of the effects of *rpoB* mutations. In this work, effects on growth, stress response and virulence of *V. vulnificus* were investigated using a set of nine spontaneous rifampicin-resistant derivatives of *V. vulnificus* CMCP6. Three different mutations (Q513K, S522L and H526Y) were identified with varying incidence rates. These three mutant types each showed high resistance to rifampicin [minimal inhibitory concentration (MIC) >800 µg ml^−1^], but different secondary effects. The strains carrying the mutation H526Y had a growth advantage in rich medium but had severely reduced salt stress tolerance in the presence of high NaCl concentrations as well as a significant reduction in ethanol stress resistance. Strains possessing the S522L mutation had reduced growth rate and overall biomass accumulation in rich medium. Furthermore, investigation of virulence characteristics demonstrated that all the rifampicin-resistant strains showed compromised motility when compared with the wild-type, but no major effects on exoenzyme production were observed. These findings reveal a wide range of secondary effects of *rpoB* mutations and indicate that rifampicin resistance is not an appropriate selectable marker for studies that aim to investigate phenotypic behaviour in this organism.

## Introduction

Antibiotic resistance is a worldwide problem and a threat to human well-being, with health, economic and social costs [1]. Rifampicin is an effective broad-spectrum antibiotic, first introduced in 1967 and currently used to treat a wide range of bacterial infections [2]. Rifampicin interferes with DNA transcription by binding the β subunit of the bacterial RNA polymerase (RNAP) [3]. In 1969, just a few years after its introduction, resistance to rifampicin was first reported [4]. In accordance with the antibacterial mode of action of rifampicin, mutations conferring rifampicin resistance map in the β subunit of RNAP, encoded by the *rpoB* gene [5–11].

Interestingly, in several microorganisms mutations conferring rifampicin resistance have been found to cause not only changes in transcription mechanisms [12–17], but also contradictory effects on cell fitness and physiology. Results of studies of *Escherichia coli* [18], *Mycobacterium tuberculosis* [19] and *Staphylococcus aureus* [20] indicated that mutations in *rpoB* can have variable influence on cell fitness, with some mutations responsible for a fitness burden and others with low or no effect on fitness. Variability of these effects typically does not correlate with the magnitude of resistance, but in *E. coli* it has been shown to be associated with modification of the mechanism of transcription and with the presence of compensatory mutations [18]. Furthermore, the effects of the same mutation can be variable in different species and depend on the genetic background or lifestyle of the resistant mutant [2, 21]. Mutations in *rpoB* conferring rifampicin resistance can also influence the physiology and survival of the bacteria. In *S. aureus* [22], *Salmonella typhimurium* [23] and *Neisseria meningitidis* [24, 25] *rpoB* mutations are responsible for changes in the physiology and metabolism, with a general effect mimicking the stringent response, and for altered expression of genes involved in virulence and survival of the bacteria in the host. These results play a fundamental role in the understanding of costs and benefits related to rifampicin resistance acquisition. In this work the effects of *rpoB* mutations conferring rifampicin resistance were addressed in the marine pathogenic bacterium *Vibrio vulnificus *.

*V. vulnificus* is a foodborne pathogen, typically found in estuarine water, which can cause severe human infections following consumption of raw or undercooked sea-food products, with subsequent septicaemia. In this type of infection, *V. vulnificus* has a fatality rate of 50 %, thus being the most fatal foodborne pathogen in the USA [26]. This organism can also cause serious wound infections, due to exposure of pre-existing wounds to seawater [27]. In this last case, the mortality rate is much lower and lethal outcomes are often linked to a pre-existing condition in the host [28]. The strain used in this work is a human clinical isolate *V. vulnificus* CMCP6 [29].

*V. vulnificus* is generally sensitive to most antibiotics [30], but cases of antibiotic resistance and multi-drug resistance have been often reported or *V. vulnificus* isolates and more generally in species of the genus *Vibrio* [31]. Rifampicin resistance has been reported in *Vibrio cholerae *, *Vibrio parahaemolyticus* [32] and *V. vulnificus* [33], but despite this, the effects of *rpoB* mutations in species of the genus *Vibrio* have been investigated rarely. Furthermore, spontaneous antibiotic resistance has wide research and biotechnological applications and it is used for genetic manipulation of several bacteria. A number of marine organisms, included members of the genus *Vibrio *, are often transformed through conjugation, and resistant strains are commonly used to optimize post-conjugation selection [34]. In *V. vulnificus* specifically, spontaneous rifampicin-resistant strains have been widely used in the past for transformation and mutagenesis protocols [35, 36], with poor consideration of the possible effects of the resistance mutation on the phenotype of the studied strains.

Due to the clinical and research relevance of rifampicin-resistant *V. vulnificus* strains, the object of this work was the investigation of effects on growth, stress survival and virulence of *V. vulnificus *. Interestingly, different *rpoB* mutations were associated with significant secondary effects on cell growth, osmotolerance, stress survival and motility of *V. vulnificus *.

## Methods

### Strains and growth conditions

The bacterial strains used in this study were *Vibrio vulnificus* CMCP6 [29] and rifampicin-resistant derivatives of this strain. Strains were grown in lysogeny broth medium with additional 0.4 M NaCl (LBN), or other concentrations where indicated. Overnight cultures were grown in 2 ml medium in 15 ml bacterial culture tubes at 37 °C with agitation. OD_595_ was measured to determine bacterial growth and biomass. All chemicals and reagents were sourced from Sigma, unless indicated otherwise.

### Generation of the rifampicin-resistant strains

*V. vulnificus* CMCP6 was cultured for 17 h in LBN at 37 °C. A 1 ml overnight culture was pelleted, resuspended in 100 µl LBN broth and spread on LBN agar containing 200 µg rifampicin ml^−1^ . After 24 h colonies of spontaneous rifampicin-resistant *V. vulnificus* were selected and streaked on LBN agar containing a lower concentration of rifampicin (100 µg ml^−1^), to confirm rifampicin resistance. Nine spontaneous rifampicin-resistant strains, derivatives of *V. vulnificus* CMCP6, were selected from two independent cultures for further study.

### Minimum Inhibitory Concentration (MIC)

To assess the rifampicin MIC, a microtiter plate was set up with twofold dilutions of rifampicin in LBN, from 800 to 3.12 µg ml^−1^, and LBN without rifampicin was used as a control. To assess the NaCl and KCl MICs, a microtiter plate was set up with twofold dilutions of NaCl or KCl in LB, from 1.6 to 0 M, and LB without additional salts was used as a blank. All the strains were grown overnight in LBN and inoculated in the microtiter plate to an initial OD of 5×10^−4^. Three biological replicates were used for each strain and each of them was assessed in two technical replicates. The plates were incubated for 24 h at 37 °C and the final OD_595_ was measured using a Sunrise microtiter plate reader.

### *rpoB* gene sequencing

To determine the mutations in the *rpoB* gene, colony PCR was performed, using each of the mentioned strains as a template. A 980 bp region of the *rpoB* gene (from nucleotide 1224 to nucleotide 2204), was amplified using *Taq* polymerase (Bioline) and using the primers rpoB_For (5′-CATTGGCCGTGATGATGCAG-3′) and rpoB_Rev (5′-TCACCACGATACGAGATGCG-3′). The amplification of the 980 bp fragment was verified through agarose gel electrophoresis, the fragment was purified using the Wizard SV Gel and PCR Clean-Up System (Promega) and sequenced by Eurofins Genomics (Ebersberg, Germany) using the same primers (rpoB_For and rpoB_Rev). To determine differences in the various *rpoB* sequences, these were aligned using the software CodonCode Aligner (CodonCode Corporation). Only mutations detected on both strands were considered to be present. The mutations were confirmed by whole-genome sequencing. Genomic DNA was extracted using the Wizard Genomic DNA Purification Kit (Promega), according to the manufacturer's protocol. Genomic DNA was sequenced by MicrobesNG (Birmingham, UK) using Illumina technology. Average read lengths were between 168 and 645 nucleotides for each sample and average fold coverage was between 52 and 166. Using BreSeq base substitution mutations were called from Read Alignment evidence using Consensus mode, with a mutation *E*-value cutoff of 10 and frequency cutoff of 0.8 (80 %). All mutations reported in Table S1 (available in the online version of this article) had a frequency >90 % and a coverage (number of reads overlapping the mutation) between 46 and 156, except for the *kefA* mutation in Rif^R^8 that had 23 reads and the VV1_2631 mutation in Rif^R^9 with 12 reads only [37].

### Growth curve

Growth was assessed for all the strains at 30 °C. The strains were grown overnight in LBN at 37 °C in biological triplicates. Bacterial cultures were then diluted in LBN to a final OD of 0.01 and assessed in three technical replicates in a microtiter plate. The plates were statically incubated in a Sunrise microtiter plate reader at the indicated temperature and the OD_595_ was measured, following 60 s of shaking, every 30 min for 24 h. The specific growth rate (μ) between two time points (t_1_ and t_2_) taken during the exponential phase was calculated using the following formula: μ=ln2/g, where g is the doubling time.

### Osmotolerance assays

To test the ability of the strains to grow in the presence of high concentrations of salts or other solutes, all strains were tested for growth ability on LB agar containing 0.8 M NaCl or KCl or 0.6 M sucrose. The strains were grown overnight in LBN at 37 °C. Bacterial cultures were then diluted to OD 1 in LBN broth and tenfold serial dilutions were performed to 10^−7^. A 3 μl sample of each dilution was spotted onto LB agar containing NaCl (0.8 M), KCl (0.8 M) or sucrose (0.6 M) and, as a control, onto LB agar with the optimal concentration of NaCl or KCl (0.4 M) and no sucrose. The plates were incubated at 30 °C and growth was evaluated after 24 and 48 h of incubation. Three biological replicates were tested for each strain in three independent experiments.

### Stress survival assays

To test survival ability in stationary growth phase, all strains were grown overnight in LBN broth at 37 °C for a minimum of 22 h. Each culture was then diluted to OD 0.1 in the appropriate medium and incubated at 30 °C for testing. The strains were tested for ethanol tolerance (10 % ethanol in LBN broth) and acidity tolerance (LBN broth adjusted with HCl to pH 4). To determine the survival, an aliquot was withdrawn at specific time points (0, 20, 40, 60 and 120 min) and used to determine the number of c.f.u. ml^−1^, through tenfold serial dilutions and plate counting. Three biological replicates were tested for each strain, each one plated in duplicate.

### Motility assay

To test motility, all strains were grown overnight in LBN broth at 37 °C. A sterilized metal wire was dipped in the overnight culture and used to pierce the centre of the motility plates (10 g tryptone, 20 g NaCl and 3.35 g agar per litre). Plates were incubated at 30 °C and the motility zone was measured after 16 h. Three biological replicates were tested for each strain.

### Exoenzyme production

To test production of haemolysin and protease, all strains were grown overnight in LBN broth at 37 °C and inoculated onto LBN plates containing specific substrates. The substrates added to the LBN agar plates were 5 % (v/v) defibrinated sheep blood (ThermoFisher Scientific) for haemolysin assay and 1 % (w/v) skim milk for protease assay. All plates were incubated at both 30 and 37 °C and clearing zones were measured after 48 h for blood agar plates and 24 h for skim milk plates.

## Results

### Three different *rpoB* mutations identified amongst nine spontaneous rifampicin-resistant strains

Nine rifampicin-resistant (Rif^R^) strains were randomly selected, as a result of two independent rifampicin-resistant generation experiments consisting of growth on agar plates with high concentrations of rifampicin (200 µg ml^−1^). The resistance of these isolates was confirmed through growth on LBN plates with 100 µg rifampicin ml^−1^ and the MIC of rifampicin for each strain (including the *V. vulnificus* CMCP6 wild-type) was assessed (Table 1). All the rifampicin-resistant strains showed growth in presence of the highest concentration tested (800 µg ml^−1^) (MIC >800 µg ml^−1^), whereas the wild-type strain was sensitive to the lowest concentration tested (3.1 µg ml^−1^) (MIC <3.1 µg ml^−1^).

**Table 1.** Growth phenotypes of *V. vulnificus* CMCP6 wild-type and rifampicin-resistant *rpoB* mutants

Isolate name	RpoB mutation	Growth rate (0.5–2.5 h) [h^−1^]*	Final OD_595_†
Wild Type	None	1.17	0.78 (0.08)
Rif^R^ 1	H256Y	1.13	0.93 (0.04)
Rif^R^ 3	H256Y	1.06	0.91 (0.02)
Rif^R^ 4	H256Y	0.97	0.90 (0.05)
Rif^R^ 5	Q513K	1.21	0.85 (0.05)
Rif^R^ 6	H256Y	0.99	0.92 (0.01)
Rif^R^ 7	Q513K	1.15	0.82 (0.03)
Rif^R^ 8	S522L	0.84	0.61 (0.08)
Rif^R^ 9	S522L	0.78	0.67 (0.03)
Rif^R^ 10	H256Y	0.92	0.90 (0.06)

*For each strain growth rate was calculated between 0.5 and 2.5 hours of growth at 30 °C

†Final OD reached after 24 hours, The reported value is the mean of the final OD of the three biological replicates, standard deviation is indicated in parentheses.

**Fig. 1.**
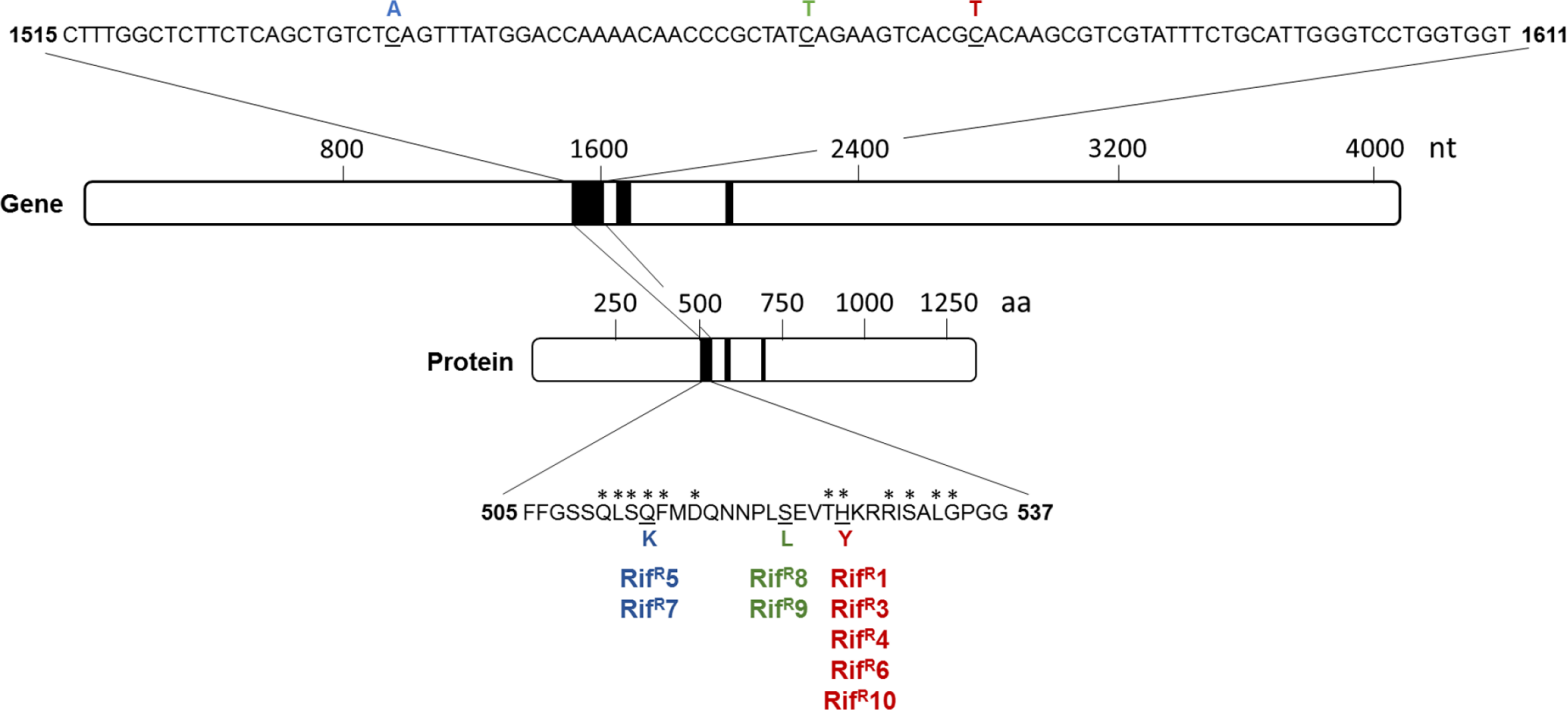
Rifampicin cluster positions and sequence in *rpoB* gene and RpoB protein. Figure adapted from [2]. The map and sequence of *V. vulnificus rpoB* gene and RNA polymerase β subunit (RpoB) and position of the rifampicin clusters I, II, III (black segments) are shown, together with mutations found in the nine rifampicin-resistant strains isolated in this work. Nucleotide mutations and corresponding amino acid changes are shown respectively at the top and bottom of the figure. Mutations and strains are grouped by colour, the H526Y mutation in red, the Q513K mutation in blue and the S522L mutation in green. Asterisks above the single amino acids indicate residues involved in direct binding to rifampicin.

The mutations conferring the Rif^R^ phenotype were mapped by sequencing the 980 bp region of the *rpoB* gene containing the three main rifampicin-binding clusters [2], the locus most frequently associated with this phenotype. Sequence alignment and comparison with the wild-type gene sequence revealed that three different *rpoB* mutations were present in the nine rifampicin-resistant *V. vulnificus* strains isolated during this work (Fig. 1). The H526Y mutation was found in five out of the nine spontaneous rifampicin-resistant strains: Rif^R^1, Rif^R^3, Rif^R^4, Rif^R^6, Rif^R^10. The H526Y mutation is known to cause a defect in transcription termination in *E. coli* [12, 13] and in *Bacillus subtilis* [16]. This mutation is frequently found in several bacterial species, due to its low effects on cell fitness [18], and this might explain the abundance of H526Y mutants amongst our rifampicin-resistant *V. vulnificus* isolates. The other two mutations, found in equal numbers, two strains each, were Q513K (Rif^R^5 and Rif^R^7) and S522L (Rif^R^8 and Rif^R^9). All these mutations map to the same rifampicin cluster, cluster I, which is close to the rifampicin binding pocket. Mutations proximal to the binding pocket generally confer a high rifampicin resistance, which explains the high MIC detected for all the nine isolates [2]. As expected, no mutations were observed in the *rpoB* gene of the wild-type strain compared with the *V. vulnificus* CMCP6 reference genome sequence (NCBI). Whole-genome sequencing (WGS) analysis was performed for the *V. vulnificus* CMCP6 WT strain and eight rifampicin-resistant strains. The results were aligned and analysed using *V. vulnificus* CMCP6 chromosome I and II sequences (GenBank: AE016795.3 and AE016796.2) as reference sequences and subsequently each rifampicin-resistant strain was compared with the WT. The WGS confirmed the *rpoB* mutations previously identified through sequencing of *rpoB* PCR products and highlighted a degree of genetic variance amongst the strains. This is not surprising as selection of the spontaneous mutants was performed through several independent experiments. The missense and nonsense non-synonymous mutations of the rifampicin-resistant strains, identified through WGS in comparison to the WT genome sequencing, are reported (Table S1). These non-*rpoB* mutations varied between the RifR strains and their presence did not correlate with the phenotypes observed.

### The S522L mutation negatively affects growth of *V. vulnificus* in an antibiotic-free environment

**Fig. 2.**
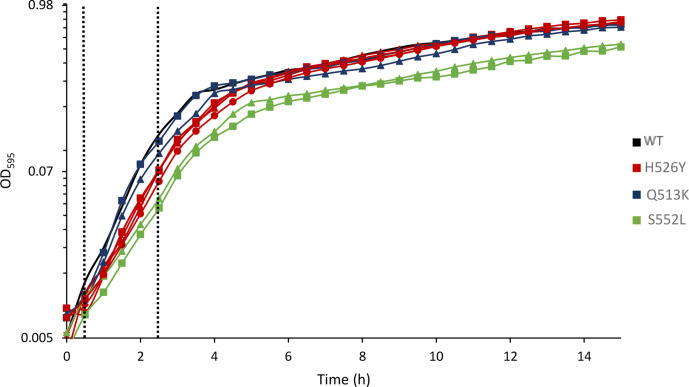
Growth curves at 30 °C in LBN broth of *V. vulnificus* CMCP6 wild-type and rifampicin-resistant *rpoB* mutants. OD_595_ was measured every 30 min for 24 h. The curves, each representing one strain, are the mean of the three biological triplicates, each assessed in three technical replicates. The strains were grouped by colour, based on *rpoB* mutation. The dotted lines represent the time interval over which the growth rate was calculated.

The rifampicin-resistant strains and *V. vulnificus* wild-type were first characterised by determining the growth of each in rich medium (LBN: LB +0.4 M NaCl) at 30 °C in the absence of rifampicin. In initial preliminary experiments growth curves at 37 and 30 °C were generated. While slight differences in growth were seen between the strains at 37 °C, the differences were more pronounced at 30 °C (data not shown). To maintain consistency throughout the study in all subsequent assays overnight cultures were grown at 37 °C to minimise differences in growth and fitness (based on our initial growth curve data) and then the actual experiments were performed at 30 °C. Growth was assessed by two parameters: growth rates during exponential phase (between 0.5 and 2.5 h of growth) and final stationary phase biomass accumulation after 24 h of growth (final OD). In antibiotic-free medium at 30 °C, the Rif^R^ strains grew with different rates during exponential phase and achieved different final biomass accumulation after 24 h of growth compared with the wild-type (Fig. 2, Table 1). The main differences in growth were observed for the S522L mutants (Rif^R^8 and Rif^R^9). These two strains showed a marked defect in growth at 30 °C, with a reduction of overall biomass accumulation and approximately 30 % reduction in growth rate, compared with the wild-type. In contrast, the five H526Y mutants showed higher overall biomass accumulation when compared with the wild-type and with the other rifampicin-resistant strains, however no differences were observed in terms of growth rate. This is consistent with the previously published descriptions of effects on cell fitness of H526Y *rpoB* mutants [18]. While significant effects on growth at 30 °C emerged for the S522L and H526Y mutants, no differences were shown for the Q513K mutants, whose growth rates and biomass accumulation were almost identical to those of the wild-type.

### The H526Y mutants have a reduction in osmotolerance

**Fig. 3.**
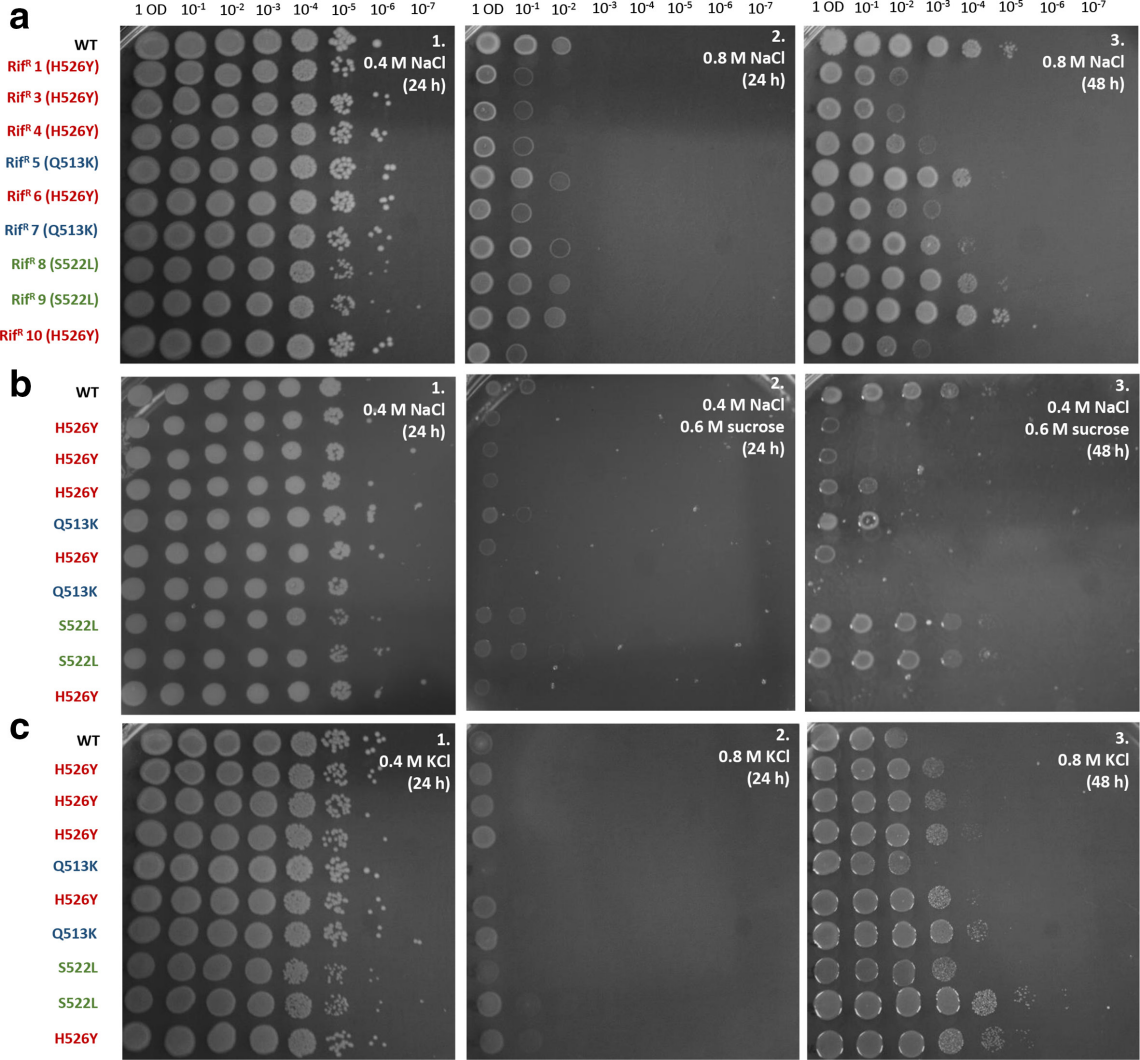
Growth assessment under osmotic stress conditions of *V. vulnificus* CMCP6 wild-type and rifampicin-resistant *rpoB* mutants. All strains were first diluted to OD_595_=1 and then tenfold serial dilutions were performed up to 10^−7^ and each dilution spotted on agar plates and incubated at 30 °C. (a) LB agar with NaCl ; (a1) LB agar with 0.4 M NaCl (LBN) after 24 h incubation; (a2) LB agar with 0.8 M NaCl after 24 h incubation; (a3) LB agar with 0.8 M NaCl after 48 h incubation. (b) LB agar with 0.4 M NaCl (LBN) and sucrose; (b1) LBN agar after 24 h incubation; (b2) LBN agar with 0.6 M sucrose after 24 h incubation; (b3) LBN agar with 0.6 M sucrose after 48 h incubation. (c) LB agar with KCl; (c1) LB agar with 0.4 M KCl after 24 h incubation; (c2) LB agar with 0.8 M KCl after 24 h incubation; (c3) LB agar with 0.8 M KCl after 48 h incubation. The *rpoB* mutations of the strains are indicated by the colours of the labels. The pictures shown are representative of three biological replicates.

**Fig. 4.**
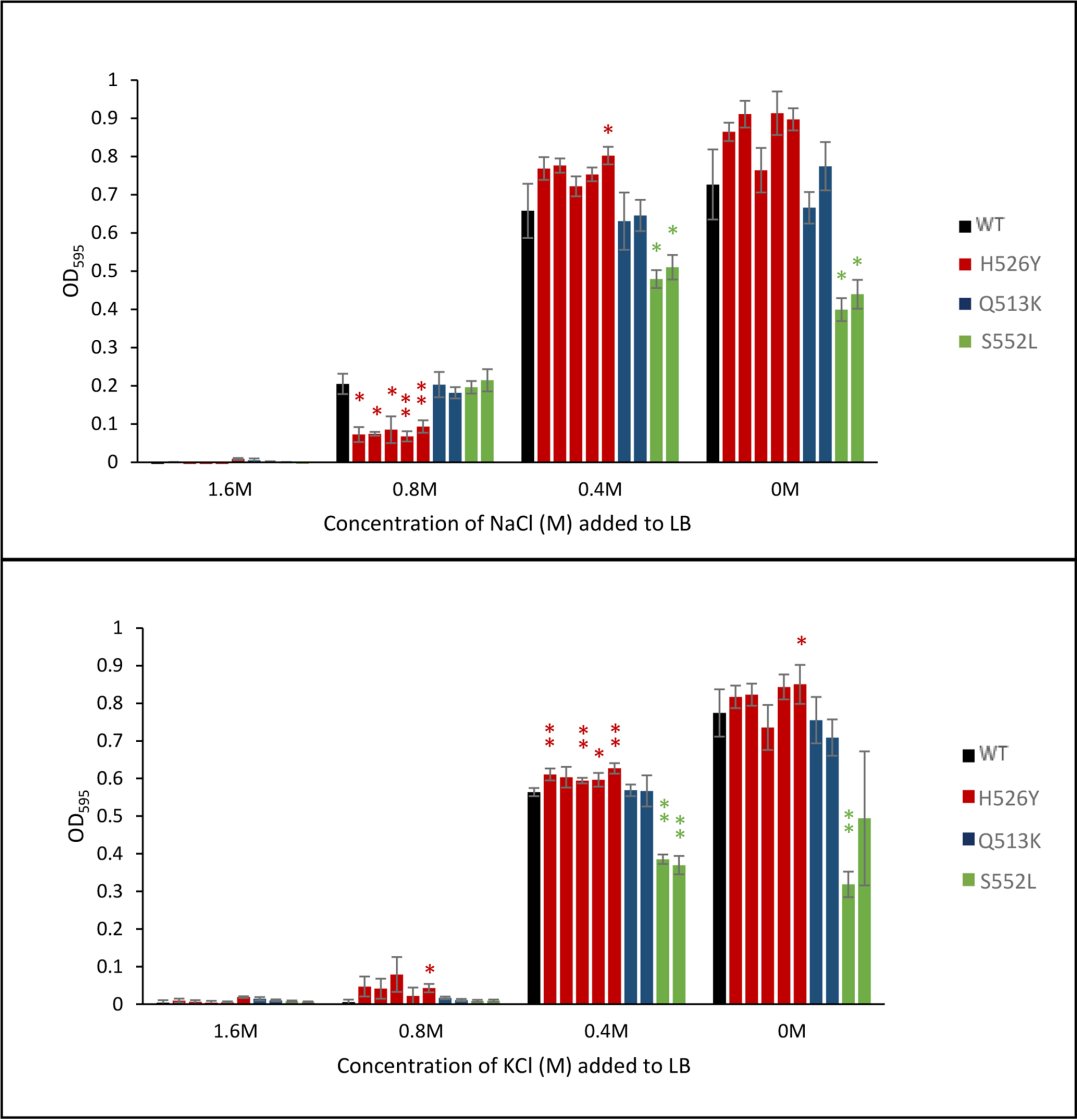
Salt tolerance of *V. vulnificus* CMCP6 wild-type and rifampicin-resistant *rpoB* mutants. OD_595_ of each strain was measured after 24 h incubation in LB broth with additional NaCl or KCl. (a) LB broth with NaCl (1.6, 0.8, 0.4, 0 M). (b) LB broth with KCl (1.6, 0.8, 0.4, 0 M). The strains were grouped on the basis of *rpoB* mutation as indicated by the colours of the bars. Three biological triplicates for each strain were tested and the reported values are the mean of the three replicates. Student's *t*-test was performed comparing mutant strains with the WT and *P* values are shown (* <0.05; ** <0.01; *** <0.001). [Note: the NaCl and KCl molarity refers to additional salt added; LB broth itself contains 10 g l^-1^ NaCl (0.16 M)].

*V. vulnificus* is a halophilic bacterium and salt concentration is known to affect the growth of this organism in the environment [28, 38], therefore possible effects of RpoB mutations on osmotolerance or salt tolerance were investigated. To determine NaCl tolerance, dilutions of all the strains were assayed for growth on LB agar with 0.6 or 0.8 M NaCl. Growth (as indicated by comparing colony or lawn formation at each dilution) was recorded after 24 h and 48 h at 30 °C (Fig. 3a). All strains grew equally well on LB and on LB with 0.6 M NaCl (Fig. 3a and data not shown). The rifampicin-resistant strains behaved variably on LB with 0.8 M NaCl, depending on the *rpoB* allele they carried. After 24 h of incubation, the H526Y mutants showed reduced growth in the presence of NaCl compared with the wild-type, while both Q513K and S522L mutants grew similarly to the wild-type strain. The phenotype was still visible after 48 h of growth. The smaller colony size of the S522L mutants at 24 h in comparison to other strains is presumably a consequence of their slower growth rate (Fig. 2), as the difference is not apparent after 48 h. To explore the possibility of a general effect of the RpoB mutation H526Y on osmotolerance of *V. vulnificus *, the same strains were tested on LB agar with 0.4 M NaCl and 0.6 M sucrose (Fig. 3b) (whose osmolarity is equivalent to 0.7 M NaCl) and LB agar with 0.6 or 0.8 M KCl (data not shown and Fig. 3c) (whose osmolarities are similar to those of to 0.6 and 0.8 M NaCl, respectively). All strains grew equally well on LB with 0.6 M KCl (data not shown). Growth in the presence of NaCl and sucrose emulated the results in the presence of 0.8 M NaCl, with reduced growth of the H526Y mutants. Surprisingly, this reduced osmotolerance was not exhibited in the presence of 0.8 M KCl. Not only did the five strains carrying the H526Y mutation not show sensitivity to KCl, but they even grew better than the wild-type in the presence of KCl. For the other four rifampicin-resistant strains, KCl tolerance was heterogeneous and not clearly correlated with the specific mutation. So, while the effects of RpoB mutations on KCl tolerance are not evident for the Q513 and S522 mutations, it is clear that the H526Y mutants have strongly reduced NaCl tolerance but not KCl tolerance, despite the similarity between these two solutes. To confirm this unexpected difference between NaCl and KCl tolerance an experiment was performed in LB broth in the presence of several concentrations of NaCl or KCl. OD endpoint measurements to quantify growth via biomass accumulation were taken after 24 h of incubation at 30 °C (Fig. 4). The increased sensitivity of the H526Y mutants (Rif^R^ 1, 3, 4, 6 and 10) to NaCl was confirmed (Fig. 4a). In LB with 0.8 M NaCl, these five strains grew significantly less than the WT and less than the other four *rpoB* mutants. In LB with 0.4 M NaCl, the same strains showed increased biomass accumulation when compared with the wild-type, which was consistent with our previous results. In addition, the behaviour in the presence of KCl was confirmed in liquid medium (Fig. 4b). At a high concentration of KCl (0.8 M) the NaCl-sensitive strains (H256Y mutants) all have a slight growth advantage, though not statistically significant, compared with the wild-type and the other *rpoB* mutants. These results demonstrate an effect of *rpoB* mutations on osmotolerance and highlight an interesting difference between NaCl and KCl response.

### The H526Y *rpoB* mutants have decreased ethanol survival

**Fig. 5.**
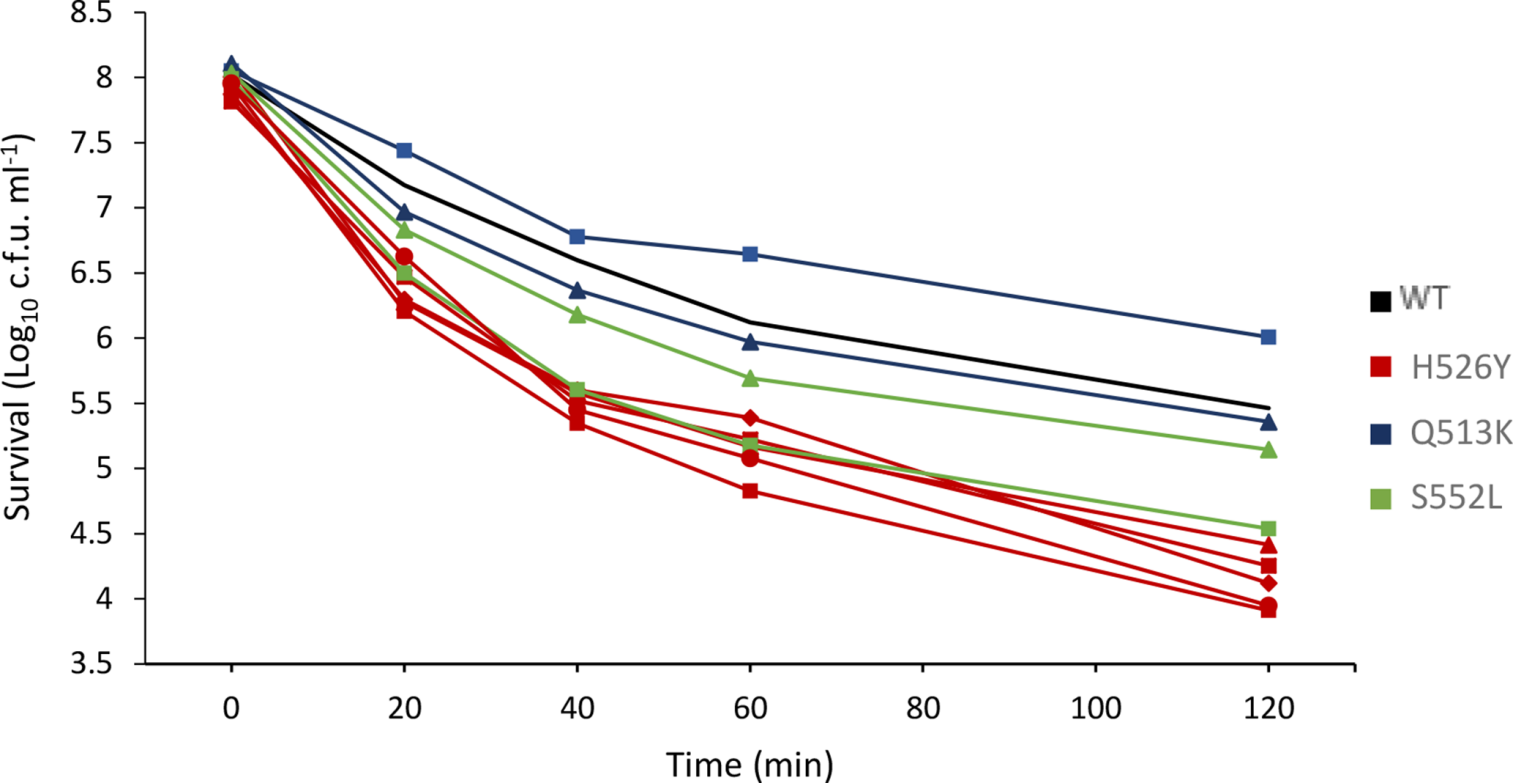
Survival assay in LBN with 10 % ethanol of *V. vulnificus* CMCP6 wild-type and rifampicin-resistant *rpoB* mutants. All strains were incubated at 30 ºC in presence of 10 % ethanol and survival was assessed, through plate counting, at five different time points. For each time point, survival was plotted as Log_10_(c.f.u. ml^−1^). The different *rpoB* mutation are indicated by the colours of the lines. Three biological triplicates for each strain were tested and the reported values are the mean of the three replicates. Student's *t*-test was performed comparing mutant strains to WT and the numbers of H526Y strains significantly different at the different time points (*P* value <0.05) were as follows: two at 20 min, four at 40 min, five at 60 min and 4 at 120 min of incubation in 10 % ethanol.

In light of this evidence, and considering the wide use of rifampicin-resistant strains of *V. vulnificus* in various studies, including stress-response research, an additional step was undertaken to investigate the effects of *rpoB* mutations on stress survival in *V. vulnificus *. The cells were tested for two stresses: ethanol (10 % ethanol in LBN) and acid (LBN pH 4). The results of the ethanol survival assay (Fig. 5) demonstrated that ethanol survival ability is affected in the H526Y mutants. All the strains carrying this mutation showed a significant reduction in survival, even after only 20 min of exposure to ethanol, compared with the wild-type strain. The same effect was not shown by the other mutants. In the acid survival assay (LBN at pH 4), all the strains (including the wild-type) showed a similar loss in viability, with some differences after 60 min of exposure but unrelated to the specific RpoB mutations (data not shown). These experiments demonstrated that that H526Y mutants of *V. vulnificus* have decreased survival in the presence of ethanol.

### *V. vulnificus rpoB* mutants have reduced motility

**Fig. 6.**
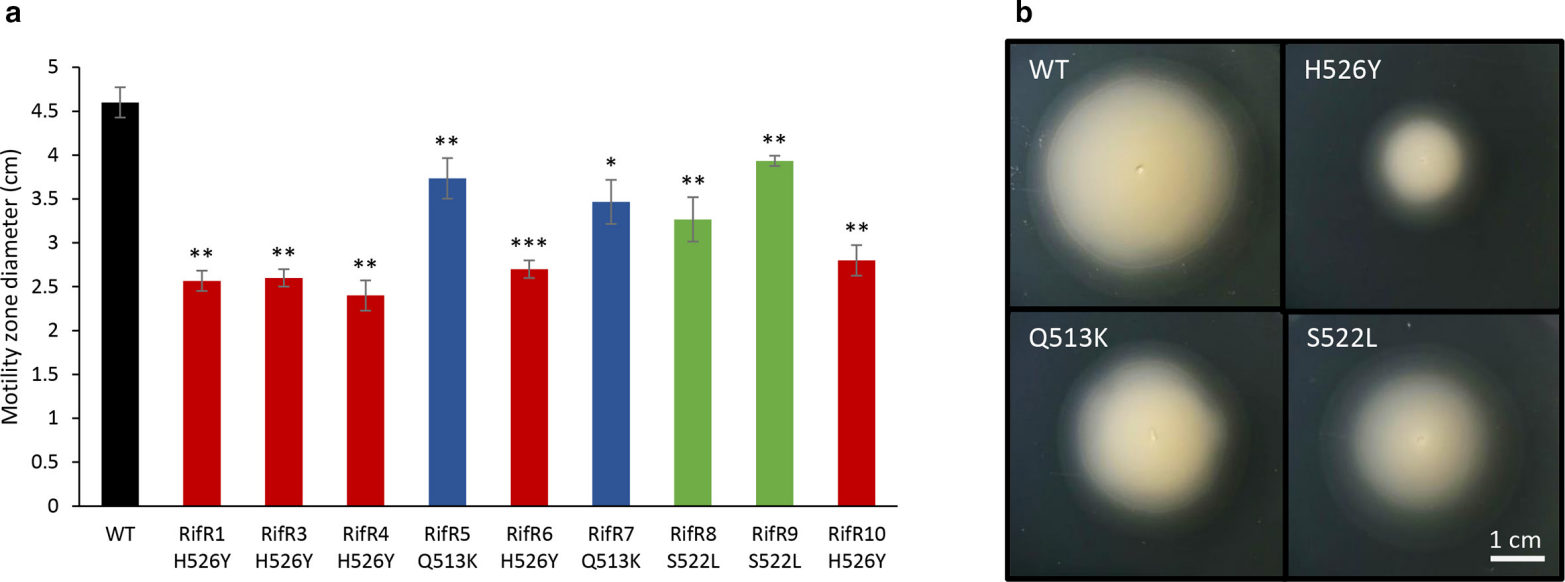
Motility assay, *V. vulnificus* CMCP6 wild-type and rifampicin-resistant *rpoB* mutants. All strains were stabbed on motility agar plates and incubated at 30 ºC. (a) The motility zone was measured after 16 h incubation. Three biological triplicates for each strain were tested and the reported values are the mean of the three replicates. Student's *t*-test was performed and *P* values are shown (* <0.05; ** <0.01; *** <0.001). The *rpoB* mutations of the strains are indicated by the colours of the bars. (b) Pictures of the motility plates were taken after 16 h incubation and one strain per each *rpoB* mutation is shown in comparison to the wild-type.

Rifampicin-resistance is a worldwide clinical emergency affecting several pathogenic organisms, including some strains of *V. vulnificus* [31, 33]. Moreover, previous work has explored the effects of RpoB mutations on virulence and infection persistence in different pathogens, including *Staphylococcus aureus* and *Mycobacterium tuberculosis* [2, 39]. To elucidate the possible effects of the three RpoB mutations studied in this work on the virulence of *V. vulnificus *, virulence characteristics were evaluated in the wild-type and in the nine rifampicin-resistant strains. All the strains were characterised for motility and exoenzyme production, both essential factors for *V. vulnificus* survival and infection in the human host [40]. Motility was studied on motility agar plates at 30 °C and the data, collected after 16 h of incubation, revealed that all the rifampicin-resistant strains were significantly less motile than the wild-type strain and, amongst all the resistant isolates, those carrying the H526Y mutation had the most compromised motility (Fig. 6). The reduced motility of the S522L mutants may be associated with their growth defect in LBN (Table 1). The strains were also tested for haemolysin and protease activity, at both 30 and 37 °C. All strains showed equal proteolytic activity on skim milk plates and haemolytic activity on blood plates at both tested temperatures, with no differences between the Rif^R^ mutants and the wild-type (data not shown). From these preliminary experiments, an involvement of *rpoB* mutations in virulence of *V. vulnificus* cannot be excluded, as the effect clearly demonstrated in this work is a general decrease in motility of all the rifampicin-resistant strains of *V. vulnificus,* and motility has been reported previously to positively affect virulence in *V. vulnificus* [41, 42].

## Discussion

Rifampicin resistance has severe effects on human health and well-being. Rifampicin resistance is caused by mutations in the *rpoB* gene, coding for the β subunit of the bacterial RNAP [5–11]. Many of these mutations have been found to affect bacterial fitness and physiology, with a wide range of secondary effects reported [18–20, 22, 24, 25, 43]. Such effects have not been investigated before in *V. vulnificus *, or in other species of the genus *Vibrio *, although rifampicin resistance has been reported in both environmental and clinical isolates of *V. vulnificus* [31, 33]. In addition, rifampicin resistance has an important biotechnological role in genetic manipulation of *V. vulnificus* and other bacteria. It is a common practice to use spontaneous rifampicin-resistant strains of *V. vulnificus* in bacterial conjugation protocols, to optimize the subsequent selection step [34]. In this work, the secondary effects of *rpoB* mutations on the growth, stress response and virulence of *V. vulnificus* were investigated.

Amongst the nine spontaneous resistant strain derivatives of *V. vulnificus* CMCP6 isolated in this work, three different *rpoB* alleles were found: the H526Y mutation was found in more than half of the strains (five strains out of nine), whereas the other two mutations, Q513K and S522L, were present in equal numbers (two strains each). WGS confirmed the *rpoB* mutations and revealed a small number of variable non-synonymous non-*rpoB* mutations (Table S1). Depending on the characteristic assessed (e.g. ethanol tolerance in Fig. 5), it is possible that the phenotypes of individual RpoB variant strains could be modulated (either tempered or intensified) due to mutations elsewhere on the genome. It is therefore important to rule out the causation of phenotypes by non-*rpoB* mutations. For this reason, throughout this study conclusions on associations of RpoB mutations with phenotypes were based on data showing clear and reproducible differences between mutant and parental strains and on evidence that all strains with the same *rpoB* mutation displayed the same phenotype.

The reults of the growth analysis in rich medium, in the absence of rifampicin, demonstrated that strains carrying different *rpoB* mutations have differential fitness and growth in an antibiotic-free environment, with a positive correlation of the H256Y mutation with biomass accumulation, a strong negative effect associated with the S522L mutation on biomass accumulation and growth rate and no effects shown by the Q513K mutants. This correlated with previous reports of variability in the effects on fitness of *rpoB* mutations in *E. coli* [18]. In the same study, the minimal effect on cell fitness of the H526Y mutation has been demonstrated, this can explain the growth advantage and the high incidence of this mutation under our experimental conditions. In addition, the possibility of a growth advantage due to *rpoB* mutation has been previously explored and demonstrated [44]. In contrast, effects on growth ability of the S522L mutation have not been demonstrated before, but previous work has shown that other mutations involving the same residue (S522F) mimic the binding of (p)ppGpp to the RNAP, thus being responsible for a stringent-like phenotype [2, 45]. This could explain the defect in growth rate and biomass accumulation observed for the S522L *V. vulnificus* mutants when compared with the wild-type and the other two rifampicin-resistant mutations.

In this work, the *V. vulnificus* H526Y mutants also showed differential salt tolerance. The mutants carrying this allele were significantly less tolerant to a high concentration of NaCl or sucrose when compared with the wild-type and the other four resistant strains. The role of RpoB mutations in osmotolerance has been previously investigated in *E. coli *, where a specific point mutation has been found to increase tolerance to a high concentration of glucose [46], however association of *rpoB* mutations with salt tolerance has never been reported before. In this study, we demonstrated that the H526Y mutants have reduced tolerance to high NaCl concentrations and high sucrose concentrations and increased tolerance to high KCl concentrations. Rif^R^6 H526Y has a D285N mutation in the potassium efflux protein KefA (Table S1), however this strain has identical KCl tolerance to the Rif^R^1, Rif^R^3 and Rif^R^4 H526Y strains, indicating that the *kefA* mutation is not responsible for, nor influences, this phenotype. These findings show an effect on osmotolerance and highlight an interesting difference between NaCl and KCl tolerance. This aspect is particularly interesting if we consider that *V. vulnificus* is a halophilic bacterium that is highly sensitive to changes in salt concentration and that this sensitivity can affect its survival and growth in the estuarine environment [28, 38]. Effects of *rpoB* mutations on stress tolerance have been previously demonstrated in *E. coli* with contrasting effects on growth under thermal stress condition, at both low and high temperature [43, 47], and mutation-specific effects on growth in low-nutrient environments [21]. In this study we demonstrated not only effects on stress tolerance but also on stress survival. In fact, we observed that the mutation H526Y also affects ethanol tolerance, with a significantly reduced survival of the H526Y mutants compared with the wild-type.

Effects of *rpoB* mutations on expression of genes involved in virulence and persistence of infection have been explored previously, with several effects observed in different microorganisms [2, 22–25, 39], generally ascribable to a stringent-like phenotype with repressed virulence and increased resistance to the host immune system, with greater infection persistence. In this work we demonstrated that all the rifampicin-resistant strains are less motile than the wild type, with a stronger effect observed for the H526Y mutants, whereas no major differences were observed in terms of exoenzyme production. Although effects on virulence of *V. vulnificus* should be further investigated, our results confirm a clear effect on motility and a possible involvement of *rpoB* mutation in virulence of *V. vulnificus* cannot be excluded, due to the previously demonstrated positive connection between motility and *V. vulnificus* virulence [41, 42].

We demonstrated effects on four main factors involved in bacterial infection: fitness, growth, stress survival and motility. These findings have an important clinical relevance, laying the basis for the analysis of costs and benefits of rifampicin resistance acquisition in *V. vulnificus* and its effects on survival and infection in the host. Of importance is also the biotechnological implication of these observations; in fact, this work indicated that the use of *V. vulnificus* rifampicin-resistant strains for genetic manipulation protocols can be disadvantageous, due to the high incidence of unpredictable secondary effects of *rpoB* mutations conferring rifampicin resistance. A valid alternative to this genetic manipulation protocol is represented by auxotrophic strains of *E. coli* that can be used for conjugation protocols and easily counter-selected in a medium lacking the required metabolite, e.g. 2,6-diaminopimelic acid (DAP) [48].

It seems reasonable to suggest that the effects of *rpoB* mutations on transcription could be the basis of the observed phenotypes. The most significant phenotype reported in this work is the effect on salt tolerance. Previous work on *E. coli* [46] indicated that osmotolerance due to *rpoB* mutations might be caused by effects on transcription of osmotic response genes, such as genes involved in compatible solute synthesis and transport. In most bacteria, including members of the genus *Vibrio *, the response to high osmolarity includes two main phases, an initial uptake of K^+^ and a subsequent accumulation of small organic molecules, through transportation and synthesis [49, 50]. On the basis of this previous knowledge, it is reasonable to speculate that in *V. vulnificus* the *rpoB* mutation might affect transcription of genes involved in osmotic tolerance, such as those involved in accumulating cytoplasmic compatible solutes. Moreover, different mutations, even if located in the same gene and in the same rifampicin cluster, could differently affect transcription with diverse secondary effects. This would explain the observed phenotypes and the differences amongst the different rifampicin-resistant strains.

In conclusion, *rpoB* mutations have secondary effects on *V. vulnificus* fitness and survival under stress conditions. Interestingly, different effects, sometimes contrasting, were observed as a result of different *rpoB* alleles. Amongst the three mutations reported, the H526Y was the one showing the strongest effects on growth, stress response and motility. These results provide significant insights into the biology of *V. vulnificus* and in particular the cost and benefits of antibiotic resistance in this marine pathogen. Moreover, they have important implications, concerning the use of rifampicin-resistant strains of *V. vulnificus* in genetic studies, especially in the field of the bacterial stress-response research.

## Supplementary Data

Supplementary material 1
