# Supplementary material for: rpoB mutations conferring rifampicin-resistance affect growth, stress response and motility in Vibrio vulnificus
Source: Microbiology (Reading). 2020 Nov 13;166(12):1160–70. doi: 10.1099/mic.0.000991 (PMC7819355; doi:10.1099/mic.0.000991)
Supplement: Supplementary material 1 [file mic-166-1160-s001.pdf]

Table S1. Whole Genome Sequencing analysis of mutations in the rifampicin-resistant strains, compared to the WT CMCP6 genome sequence.

**Gene**	**Mutation**	**Description**	**Rif^{R}1**	**Rif^{R}3**	**Rif^{R}4**	**Rif^{R}5**	**Rif^{R}6**	**Rif^{R}7**	**Rif^{R}8**	**Rif^{R}9**
VV1_0007	D285N	Potassium efflux system *kefA*					X			
mviN	A158V	integral membrane protein MviN							X	
gspD	G489E	general secretion pathway protein D			X					
rpoB	Q513K	DNA-directed RNA polymerase, beta subunit				X		X		
rpoB	S522L	DNA-directed RNA polymerase, beta subunit							X	X
rpoB	H526Y	DNA-directed RNA polymerase, beta subunit	X	X	X		X			
VV1_1376	T690K	Multimodular transpeptidase-transglycosylase			X					
recA	G179S	protein RecA							X	
VV1_1668	D524N	Glucosamine-link cellobiase					X			
VV1_1763	M3I	Putative isomerase						X		
VV1_1999	R220H	Response regulator	X	X						
VV1_2609	G224E	Universal stress protein E				X				
VV1_2631	H340Y	hypothetical protein							X	X
VV2_0134	C236Y	Decaheme cytochrome c MtrA			X					
VV2_0149	R334H	Arylsulfatase			X	X	X	X	X	X
clpV	V115I	type VI secretion ATPase, ClpV1 family	X	X						
VV2_0556	H43D	Predicted signal transduction protein	X	X						
VV2_0714	R337C	YjeF protein				X				
VV2_0774	W195*	Amino acid transporter								X
